# Exercise training improves liver steatosis in mice

**DOI:** 10.1186/s12986-015-0026-1

**Published:** 2015-08-07

**Authors:** Sheril Alex, Andreas Boss, Arend Heerschap, Sander Kersten

**Affiliations:** Nutrition, Metabolism and Genomics Group, Wageningen University, Stippeneng 4, 6708 WE Wageningen, The Netherlands; Department of Radiology, Radboud University Medical Center, Geert Grootplein 10, 6500 HB Nijmegen, The Netherlands

## Abstract

**Background:**

Non-alcoholic fatty liver disease (NAFLD) is rapidly turning into the most common liver disorder worldwide. One of the strategies that has been shown to effectively improve NAFLD is regular exercise, which seems to lower steatosis partly independent of weight loss. However, limited data are available about the mechanisms involved. The aim of the present study was to identify the mechanisms underlying the effect of regular exercise on liver steatosis.

**Methods:**

Non-obese male mice were rendered steatotic by feeding a sucrose-enriched choline-deficient diet. They were then subjected to daily treadmill running for three weeks, whereas the control mice remained sedentary.

**Results:**

Compared to the untrained mice, trained mice showed similar adipose tissue mass but had significantly reduced size of lipid droplets in the liver coupled with a reduction in liver triglyceride content (~30 %, *P* < 0.05). Levels of various plasma lipid parameters and plasma glucose were similar between the trained and untrained mice, whereas levels of hepatic glycogen were significantly higher in the trained mice. Hepatic triglyceride secretion rate and de novo lipogenesis were unchanged between the two sets of mice, as were indicators of lipolysis and autophagy. Finally, whole genome expression profiling indicated a mild stimulatory effect of exercise training on PPARα-mediated regulation of oxidative metabolism, including fatty acid oxidation.

**Conclusions:**

Taken together, our study suggests that the lowering of hepatic steatosis by repeated exercise is likely due to activation of fuel oxidation pathways in liver.

## Background

Non-alcoholic fatty liver disease (NAFLD) is one of the most common liver disorders worldwide. NAFLD is frequently paralleled by obesity and is often referred to as the hepatic manifestation of the metabolic syndrome [1]. The main initial feature of NAFLD is hepatic steatosis, defined by hepatic lipid levels in excess of 5 % of liver weight, which can further progress to steatohepatitis, hepatic fibrosis and cirrhosis [2].

The liver is the central regulator of triglyceride and carbohydrate metabolism. Excess storage of lipids in the liver can be caused by a number of metabolic derangements including defective fatty acid oxidation, enhanced lipogenesis, impaired triglyceride secretion and increased uptake of fatty acids from the circulation [3]. In humans, hepatic steatosis is often accompanied by insulin resistance. Evidence abounds that insulin resistance promotes a fatty liver, while at the same time a fatty liver may lead to hepatic insulin resistance, potentially causing a vicious cycle [4]. It is generally believed that enhanced hepatic fatty acid influx originating from either the diet or adipose tissue lipolysis is the primary driver for development of NAFLD during obesity and insulin resistance [5, 6], although alterations in other metabolic pathways including enhanced lipogenesis likely contribute as well.

Apart from weight loss induced by hypocaloric diets (possibly preceded by bariatric surgery), current treatment options for NAFLD are limited. With respect to the pharmacological treatment of NAFLD, the farnesoid X receptor agonist obetocholic acid recently showed promise for the treatment of steatohepatitis, but its safety profile needs further evaluation [7]. A similar case can be made for the dual Peroxisome Proliferator Activated Receptor α/γ agonist GFT505 [8]. An alternative remedy with a good safety record and capable of improving multiple cardio-metabolic risk factors including NAFLD is physical exercise [9]. A number of recent human trials have demonstrated a beneficial effect of regular exercise on NAFLD [10–13]. While part of the improvement in NAFLD by exercise may be related to exercise-induced weight loss, the results of a recent meta-analysis indicate that the benefits of regular exercise on liver fat occur with minimal or no weight loss [14].

Efforts are underway to better understand the physiological mechanisms underlying exercise-mediated improvement in NAFLD, primarily via the use of appropriate animals models [15]. Nearly all studies have relied on animal models of NAFLD that are characterized by obesity. In those studies, improvements in liver steatosis following exercise training are often secondary to a reduction in body weight [16–21]. Limited data are available from animal models that exhibit a reduction in liver fat by exercise training without significant weight loss [22, 23]. Accordingly, the full scope of the exercise-induced effects on steatosis and hepatic lipid metabolism remains unclear. The aim of the present study was to gain more insight into the mechanisms underlying the direct effect of exercise training on liver steatosis using a non-obese mouse model of NAFLD. To this end, C57BL/6 mice rendered steatotic by feeding a diet rich in sucrose but deficient in choline (sucrose-enriched choline-deficient diet, SECDD) were used for a treadmill-based exercise training program. Our study results show that exercise training reduced liver fat accumulation in the non-obese mice, possibly due to the activation of fuel oxidation pathways in liver.

## Materials and methods

### Mice model of fatty liver and exercise training protocol

The non-obese mouse model of fatty liver used in this study involved feeding male C57BL/6 mice a low-fat, high-sucrose, choline-deficient semi-synthetic diet referred to as sucrose-enriched choline-deficient diet (SECDD) for five weeks. The composition of the SECDD is shown in Table 1. Before proceeding to the main study, a pilot experiment using C57BL/6 mice was conducted to examine the effectiveness of SECDD diet in inducing liver steatosis in comparison with feeding regular chow.

**Table 1:** Composition of the sucrose enriched choline deficient diet (SECDD)

	SECDD
Kcal/g	3.8
Protein (Kcal %)	18.5
Carbohydrate (Kcal %)	70.6
Fat (Kcal %)	10.8
Ingredients (g/Kg)	
Casein	170
L-Cystine	3
Corn starch	125
Maltodextrin	100
Sucrose	425
Cellulose	50
Soybean oil	25
Palm oil	20
Mineral-salt mix	45
Vitamin Mix	10
Choline bitartarate	0

For the main study, male C57BL/6 mice were fed the SECDD for five weeks, after which the mice were divided into an exercise (trained) and a sedentary (untrained) group. Mice belonging to the trained group did a treadmill-based running exercise (TSE PhenoMaster, TSE Systems, Germany) for a period of 3 weeks; 5 days/week, 1 h/day. In the first week, mice ran at 14 m/min with 0° incline and in the following weeks they ran at a speed of 16 m/min with 2° incline, representing moderate intensity exercise for the mice. During the exercise training period, the mice continued to be fed on SECDD. The untrained group of mice were placed on a non-moving treadmill also for a period of 3 weeks; 5 days/week, 1 h/day. Body weight and food intake were measured weekly throughout the experiment. After the last bout of exercise, the mice were returned to their cage and 24 h later, a subset of mice from the trained and untrained groups were either used to study *in-vivo* hepatic triglycerides secretion (*n* = 7 untrained, *n* = 9 trained), sacrificed to collect tissues for biochemical and histological study (*n* = 7 untrained, *n* = 9 trained) or sacrificed to collect tissues for various measurements, including hepatic de novo lipogenesis (DNL) (*n* = 12 untrained, *n* = 12 trained). A number of measurements were performed in all mice within the last two cohorts, raising the number of biological replicates to 19 or 21 per group. Prior to sacrifice, mice were put under anesthesia using isoflurane and blood was drawn via orbital puncture into EDTA-containing tubes. Plasma was prepared by centrifugation at 4 °C and stored at −80 °C. All animal experiments were approved by the animal welfare committee of Wageningen University.

### Plasma and tissue biochemical measurements

Plasma samples of non-fasted mice were analysed for glycerol (Diasys, Germany), triglycerides (Liquicolor, Germany), non-esterified fatty acids, NEFA (Wako Chemicals, Germany), cholesterol (Diasys, Germany) and alanine aminotransferase, ALT (Abcam, UK) using commercially available kits according to manufacturer’s instructions. Tissue glycogen levels were measured according to a method described previously [24]. Briefly, approximately 50 mg of tissue was digested using 1 N NaOH at 80 °C for 15 min. Glycogen was precipitated from the supernatant by adding 100 % ethanol at 2:1 v/v and the precipitate was pelleted down. Pellet was washed with 80 % ethanol and solubilised in water by incubating at 37 °C for 15 min. The glycogen sample was then digested to glucose by incubating at 42 °C for 2 h with amyloglucosidase (Sigma, USA) in 0.2 M sodium acetate buffer (pH 4.8). The amount of glucose in the digested samples was measured using a glucose assay kit (DiaSys, Germany).

Liver triglycerides were determined in 5 % liver homogenates prepared in buffer containing 250 mm sucrose, 1 mm EDTA, 10 mm Tris–HCl (pH 7.5) using a commercially available kit (Liquicolor, Germany). Histological analysis of liver morphology and lipid content was done using haematoxylin-eosin (H&E) and oil-red O (ORO) staining.

### De-novo hepatic VLDL triglyceride secretion and lipogenesis

For the VLDL triglyceride secretion test, mice fasted for 4 h were injected via the orbital plexus with 500 mg/kg bodyweight of the lipoprotein lipase inhibitor Triton WR1339 (Tyloxapol) as 15 % solution under general anesthesia. Blood was collected by tail bleeding every 30 min for 2.5 h as the mice remained sedated. Plasma triglyceride levels were measured in the blood samples collected at different time points using an enzymatic kit (Liquicolor, Germany). Glucose was measured in the baseline plasma sample (Diasys, Germany).

The rate of liver *de novo* lipogenesis (DNL) was measured by ^2^H NMR using ^2^H enrichment of liver triglycerides [25]. At the end of the last exercise training session, a set of mice belonging to both trained and untrained groups were rested for 5-6 h and given an intraperitoneal injection of 99.9 % ^2^H_2_O (Sigma-Aldrich, USA) as saline. The mice were then returned to their cage and had free access to food and water. In order to have constant body water enrichment, the drinking water was enriched with ^2^H_2_O at 3 %. They were then sacrificed after 16 h, and blood samples were obtained for the determination of plasma ^2^H-enrichment. The livers were excised and hepatic lipids extracted [26]. The extract was dissolved in chloroform and a mixture of ^1^H/^2^H-pyrazine was added as reference. Proton-decoupled ^2^H NMR spectra (WALTZ 16-decoupling, 90° hard pulse for excitation) and ^1^H-spectra of lipids were acquired (Bruker Avance III 500 MHz) and the methyl-group signals of the ^1^H-and ^2^H-spectra were quantified relative to the signal of the respective standard. The ^2^H-enrichment of body-water was determined as previously described [27]. The ratio between the ^2^H-enrichment of the methyl-groups of hepatic triglycerides and body-water represents the fractional contribution of DNL to hepatic triglycerides.

### Hepatic gene and protein expression levels

Total RNA was isolated from the mouse tissues using TRIzol reagent (Invitrogen, Breda, The Netherlands). RNA was reverse transcribed using RevertAid First strand cDNA synthesis kit (Thermoscientific). Real-time PCR was carried out using SensiMiX (Bioline) on a CFX 384 Bio-Rad thermal cycler (Bio-Rad). 36B4 was used as housekeeping gene. Primers sequences used are shown in Table 2.

**Table 2:** List of primers

Gene	Forward primer	Reverse primer
Scd1	TAGCCTGTAAAAGATTTCTGCAAACC	CCGGAGACCCTTAGATCGA
Fasn	TCCTGGGAGGAATGTAAACAGC	CACAAATTCATTCACTGCAGCC
Gpam	ACAGTTGGCACAATAGACGTTT	CCTTCCATTTCAGTGTTGCAGA
CD36	TCCAGCCAATGCCTTTGC	TGGAGATTACTTTTCAGTGCAGAA
Fabp2	AAAGGAAACCTCATTGCCACCA	AATGTCGCCCAATGTCATGGTA
Mttp	ATACAAGCTCACGTACTCCACT	TCCACAGTAACACAACGTCCA
Nr1h3	GCT CTG CTC ATT GCC ATC AG	TGTTGCAGCCTCTCTACTTGGA
Acaca1	GCCATTGGTATTGGGGCTTACC	CCCGACCAAGGACTTTGTTG
Srebf1	GGAGCCATGGATTGCACATT	CCTGTCTCACCCCCAGCATA
Mlxipl	CTGGGGACCTAAACAGGAGC	GAAGCCACCCTATAGCTCCC
Slc2a2	TCAGAAGACAAGATCACCGGA	GCTGGTGTGACTGTAAGTGGG
Lipe	TCAACCGACCAGCAGTGCTC	CTCTGGGTCTATGGCGAATC
Pnpla2	CAACGCCACTCACATCTACGG	GGACACCTCAATAATGTTGGCAC
Lipa	TGTTCGTTTTCACCATTGGGA	CGCATGATTATCTCGGTCACA
Atg3	ACACGGTGAAGGGAAAGGC	TGGTGGACTAAGTGATCTCCAG
Atg5	AGCCAGGTGATGATTCACGG	GGCTGGGGGACAATGCTAA
Sqstm1	AGGATGGGGACTTGGTTGC	TCACAGATCACATTGGGGTGC
Map1lc3b	TTATAGAGCGATACAAGGGGGAG	CGCCGTCTGATTATCTTGATGAG
Il-1b	CAGGCAGGCAGTATCACTCA	AGGTGCTCATGTCCTCATCC
36b4	ATGGGTACAAGCGCGTCCTG	GCCTTGACCTTTTCAGTAAG

Tissue homogenates were made using RIPA buffer with added protease inhibitor cocktail and spun to pellet the cell debris. Supernatant was collected and the protein concentration determined using the BCA method (Pierce BCA protein assay kit). Equal amount of protein was subjected to a 4-15 % gradient gel electrophoresis and immunoblotted using the following antibodies for Tubulin (1:1000, SantaCruz), GAPDH (1:1000, Santa Cruz), LC3 and p62 (1:1000, Novus biologicals); AMPK, pAMPK, Cytochrome C (1:1000, Cell signalling technology). Blots were further incubated with the appropriate HRP conjugated secondary antibody (1:5000) and developed using BioRad Clarity (BioRad, USA). The bands were visualised using ChemiDoc system (BioRad, USA) and quantified with Image Lab software.

### Micro-array analysis

After TRIzol, RNA was further purified using RNeasy micro columns (Qiagen, Venlo, the Netherlands). RNA integrity was checked on an Agilent 2100 bioanalyzer (Agilent Technologies, Amsterdam, the Netherlands) using 6000 Nano Chips according to the manufacturer’s instructions. Purified RNA (100 ng) was labeled with the Ambion WT expression kit (Invitrogen) and hybridized to an Affymetrix Mouse Gene 1.1 ST array plate (Affymetrix, Santa Clara, CA). Hybridization, washing, and scanning were carried out on an Affymetrix GeneTitan platform according to the instruction by the manufacturer. Arrays were normalized using the Robust Multiarray Average method [28, 29]. Probe sets were defined according to Dai et al. [30]. In this method probes are assigned to Entrez IDs as an unique gene identifier. The P values were calculated using an Intensity-Based Moderated T-statistic (IBMT) [31]. The microarray data were submitted to the Gene Expression Omnibus (accession number pending). Gene set enrichment analysis (GSEA) was used to find enriched gene sets in the induced or suppressed genes [32]. Genes were ranked based on the IBMT-statistic and subsequently analyzed for over- or underrepresentation in predefined gene sets derived from Gene Ontology, KEGG, National Cancer Institute, PFAM, Biocarta, Reactome and WikiPathways pathway databases. Only gene sets consisting of more than 15 and fewer than 500 genes were taken into account. Statistical significance of GSEA results was determined using 1000 permutations.

### Statistical analysis

All the results are expressed as mean ± SEM. Comparisons were made between the trained and untrained mice. Statistical significance was tested using a two-tailed Student’s *t*-test and *p* < 0.05 was considered as significantly different.

## Results

### Exercise training reduced liver fat in SECDD-fed steatotic mice

**Fig. 1:**
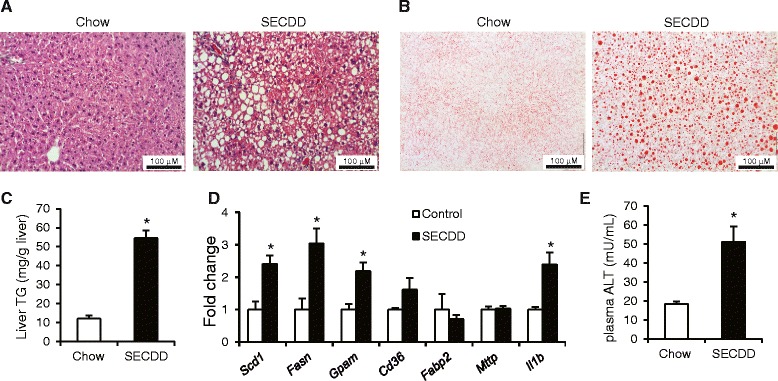
Elevated liver triglycerides in mice fed SECDD. **a** Hematoxilin & Eosin staining and **b** oil red O staining of representative sections of liver from male C57BL/6 mice fed chow or SECDD for five weeks. **c** Liver triglycerides. **d** Expression of selected genes as determined by qPCR. E) Plasma alanine aminotranferase activity. Error bars represent standard error (*n* = 4–6). Asterisk indicates statistically significant according to Student’s *t*-test (*P* < 0.05)

We employed a non-obese steatosis model, 1) to avoid the possible interference of obesity on the ability of the mice to run, 2) to lower the chance of confounding by exercise-induced weight loss. The non-obese steatosis model consisted of five weeks of feeding C57BL/6 mice a diet enriched in sucrose but deficient in choline (sucrose-enriched choline-deficient diet, SECDD). Hematoxilin & eosin staining (Fig. 1a) and oil red O staining (Fig. 1b) indicated increased number and size of lipid droplets in livers of mice fed the SECDD. Biochemical analysis showed nearly six-fold higher liver triglyceride levels in the SECDD-fed mice as compared with mice fed chow (Fig. 1c). Gene expression analysis by qPCR showed significant upregulation of a limited number of genes involved in hepatic lipogenesis, including stearoyl-CoA desaturase (*Scd1)*, fatty acid synthase (*Fasn*) and Glycerol 3-phosphate acyltransferase (*Gpam)* in the mice fed SECDD (Fig. 1d). Expression of the inflammatory marker Interleukin-1β (*Il1b*) was also increased. Consistent with the steatotic phenotype, plasma ALT levels were increased three-fold in mice fed SECDD (Fig. 1e). Taken together, SECDD feeding serves as an appropriate model to induce hepatic steatosis without high-fat overfeeding.

**Fig. 2:**
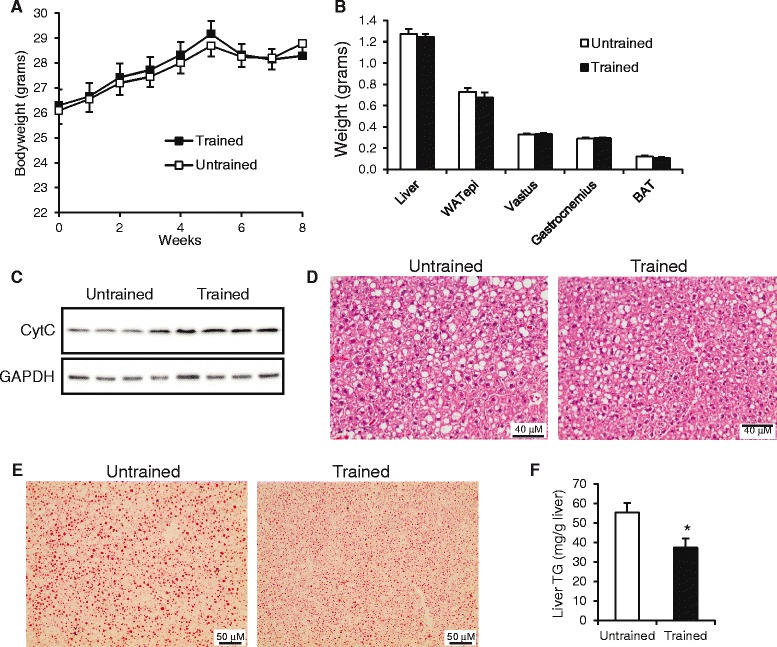
Decreased liver triglycerides in trained mice. **a** Bodyweight changes in the trained and untrained (sedentary) mice. Daily treadmill exercise was started at week 5. **b** Weight of liver, epididymal fat depot (WATepi), vastus lateralis muscle, gastrocnemius muscle, and interscapular brown adipose depot (BAT). **c** Immunoblot of cytochrome C in gastrocnemius muscle. **d** Hematoxilin & Eosin staining of representative liver sections. **e** Oil red O staining of representative liver sections. **f** Liver triglycerides measured enzymatically. Error bars represent standard error (*n* = 7–21). Asterisk indicates statistically significant according to Student’s *t*-test (*P* < 0.05)

To study the effect of endurance exercise training on liver fat content, mice rendered steatotic by SECDD feeding were subjected to a three week exercise regimen consisting of one hour of treadmill running daily. Sedentary (untrained) mice were placed on a non-moving treadmill lane for one hour daily. Twenty-four hours after the last exercise bout, the mice were subjected to different types of measurements or sacrificed. Final bodyweight and bodyweight gain were not different between the trained and untrained mice (Fig. 2a). Also, liver weight, weight of the epididymal fat pad, brown adipose tissue weight, and weights of vastus and gastrocnemius muscle were not different between the trained and untrained mice (Fig. 2b). As expected, exercise training was associated with a noticeable increase in cytochrome C content in gastrocnemius muscle (Fig. 2c).

**Fig. 3:**
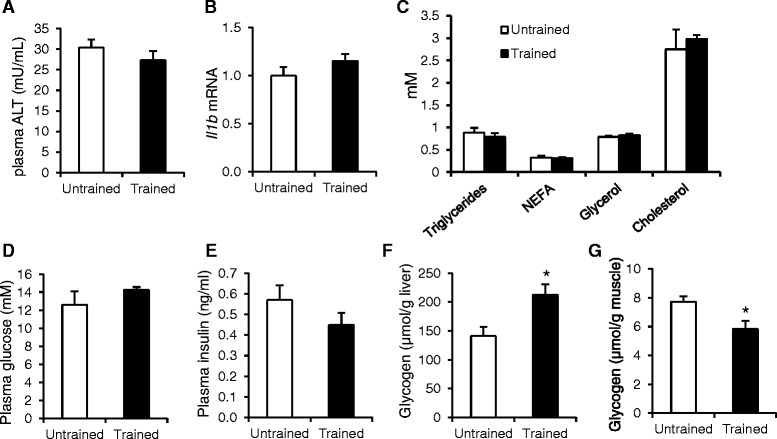
Metabolic parameters in trained and untrained mice. **a** Plasma alanine amino transferase activity. **b** Hepatic gene expression of Interleukin-1β. **c** Plasma concentration of lipid parameters. **d** Fasting plasma glucose concentration. **e** Plasma insulin concentration. **f** Liver glycogen content. **g** Muscle (gastrocnemius) glycogen content. Error bars represent standard error (*n* = 7–20). Asterisk indicates statistically significant according to Student’s *t*-test (*P* < 0.05)

Strikingly, staining of liver sections by hematoxilin & eosin (Fig. 2d) and oil red O staining (Fig. 2e) showed fewer and smaller lipid vacuoles in the liver of the trained mice as compared with the untrained mice. The reduced lipid accumulation in the livers of the trained mice was supported by oil red O staining and biochemical analysis, which showed an approximate 30 % reduction in liver triglyceride content (*P* < 0.05; Fig. 2f). Despite the reduced hepatic steaotosis following training, plasma ALT levels (Fig. 3a) and hepatic expression of inflammatory marker *ll-1β* (Fig. 3b) were not affected by exercise training. Furthermore, exercise training did not affect plasma triglyceride, free fatty acids, glycerol or cholesterol levels (Fig. 3c). Plasma glucose and insulin levels also remained unchanged (Fig. 3d and e). Interestingly, liver glycogen levels were significantly higher in the trained mice as compared with the untrained mice (Fig. 3f), whereas the opposite pattern was observed for muscle glycogen levels (Fig. 3g).

**Fig. 4:**
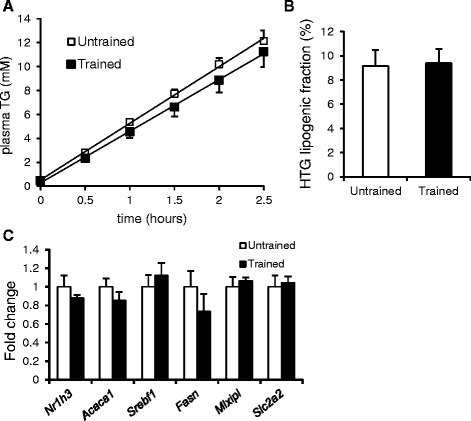
Hepatic triglyceride production and de novo lipogenesis in trained and untrained mice. **a** Plasma triglyceride concentrations in trained and untrained mice after injection with tyloxapol. **b** Percentage of hepatic triglycerides derived from de novo lipogenesis. **c** Expression of selected genes as determined by qPCR. Error bars represent standard error (*n* = 7–12)

The reduction in liver triglycerides in the trained mice may be explained by a number of mechanisms including enhanced hepatic triglyceride secretion, decreased hepatic lipogenesis, enhanced intracellular lipolysis, and increased hepatic fatty acid oxidation. To differentiate between these possibilities, we first determined the effect of exercise training on hepatic triglyceride secretion via injections with tyloxapol. No significant difference in the rate of increase in plasma triglyceride levels following tyloxapol injection was observed between trained and untrained mice (Fig. 4a), indicating that hepatic triglyceride secretion was unaffected by exercise training. Next we studied the contribution of *de novo* lipogenesis towards the liver triglyceride pool using deuterium-labeled water. It was observed that in the untrained mice about 9 % of hepatic triglycerides were derived from hepatic *de novo* lipogenesis. The same number was found in trained mice, suggesting that exercise training did not alter the rate of *de novo* lipogenesis (Fig. 4b). qPCR analysis also did not reveal changes in expression of relevant lipogenic genes (Fig. 4c).

**Fig. 5:**
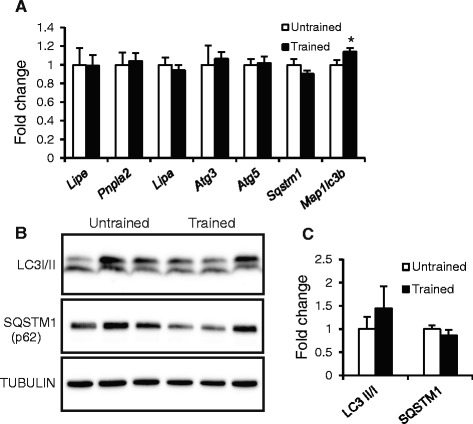
Effect of training on lipolysis and autophagy. **a** Expression of selected genes in liver as determined by qPCR. **b** Immunoblot of LC3I/LC3II and p62. **c** Quantification of immunoblots. Error bars represent standard error (*n* = 4–12). Asterisk indicates statistically significant according to Student’s *t*-test (*P* < 0.05)

To investigate whether exercise training may affect intracellular lipolysis, we measured expression of genes involved in lipolytic pathways, including Lipe (hormone sensitive lipase), Pnpla2 (adipose triglyceride lipase), and Lipa (lysosomal lipase), as well as the markers of autophagy such as microtubule-associated protein 1 light chain 3 beta Map1lc3b (*LC3b*), sequestosome 1 (*Sqstm1/p62*), autophagy-related 3 (*Atg3*) and *Atg5* (Fig. 5a). None of these genes was differentially expressed between trained and untrained mice, with the exception of Map1lc3b (*LC3b*), which was slightly but significantly higher in the trained mice. Levels of Sqstm1 protein and the relative abundance of LC3I and LC3II, which is used as marker for activated autophagy, were similar between the two groups (Fig. 5b and c).

**Fig. 6:**
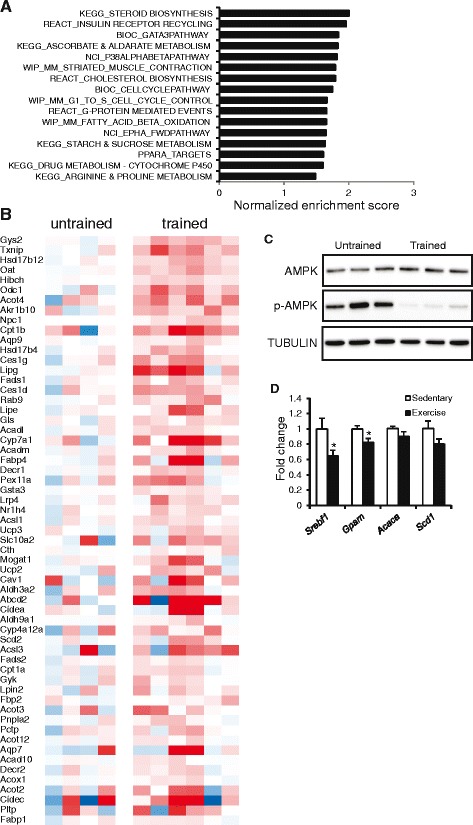
Exercise training altered liver oxidative processes. **a** Gene sets positively enriched in the livers of trained mice as compared to untrained mice ranked according to normalized enrichment score (gene set enrichment analysis). The enrichment score reflects the degree to which a gene set is overrepresented at the top or bottom of a ranked list of genes. Normalization accounts for differences in gene set size and in correlations between gene sets and the expression dataset. The normalized enrichment score is most often used to compare analysis results across gene sets. **b** Gene expression changes in liver illustrated by heat map of positively enriched genes belonging to the gene set PPAR targets. **c** Immunoblot of AMPK and phospho-AMPK. **d** Expression of selected genes in liver of mice after 90 min on a non-moving treadmill or a running treadmill (12 m/min) [42], as determined by qPCR. Asterisk indicates statistically significant according to Student’s *t*-test (*P* < 0.05)

To further investigate the effect of exercise training in mouse liver, we performed microarray analysis. To determine which pathways were affected by exercise training we performed gene set enrichment analysis. The analysis revealed significant induction of a number of pathways, as shown in Fig. 6a. Interestingly, genes within the gene set “fatty acid beta oxidation” and “PPARα targets” were significantly enriched in the livers of trained mice as compared to the untrained mice. Although PPARα expression itself was not upregulated, multiple genes that are under its transcriptional control were modestly induced in the trained mice (Fig. 6b). These data are suggestive of relatively weak induction of PPARα-dependent genes and pathways, including fatty acid oxidation. To test whether mitochondrial metabolism in liver is activated by exercise training, we measured activation of AMP-activated protein kinase (AMPK). We reasoned that if mitochondrial metabolism is elevated in the trained animals, ATP levels may be higher in the resting state leading to lower activation of AMPK. Consistent with this notion, it was found that pAMPK was markedly lower in trained mice as compared to the untrained mice (Fig. 6c).

An additional explanation for the reduction in liver triglycerides by exercise training might be that every single exercise bout causes a minor decrease in the liver triglyceride pool, cumulatively leading to the 30 % reduction in hepatic triglyceride levels after 3 weeks of exercise training. Such a minimal reduction in liver triglyceride levels by a single exercise bout would be impossible to demonstrate experimentally, but may be indirectly deduced from induction or repression of certain metabolic pathways. Indeed, a single 90 min exercise bout in mice significantly reduced hepatic expression of the lipogenic transcription factor Sterol regulatory element binding protein 1 (*Srebf1*) and its target *Gpam* (Fig. 6d).

## Discussion

In the current study, we investigated the effect of exercise training on liver triglyceride levels using a non-obese mouse model of steatosis. It was observed that a treadmill based exercise training for three weeks reduced liver fat accumulation without causing any changes in adipose tissue mass. Theoretically, a reduction in liver triglyceride levels can be caused by a number of different mechanisms, including decreased hepatic lipogenesis and increased triglyceride secretion. In our study, exercise training did not affect *de novo* lipogenesis, nor did it affect hepatic triglyceride secretion. Most of the fatty acids that serve as substrates for hepatic triglycerides are derived from circulating free fatty acids. In our study we did not observe any sustained changes in plasma free fatty acid levels. Unfortunately, we were unable to directly determine free fatty acid uptake and fluxes into the liver. Further, we did not find strong indications that lipophagy, describing the breakdown of intracellular lipids via autophagy [33], was altered in the trained mice. Instead, our data suggest that exercise training is associated with mild activation of PPARα-mediated regulation of oxidative metabolism in liver at rest, which may account for the observed reduction in liver triglycerides. It should be noted that the magnitude of upregulation of genes involved in beta oxidation as well as other PPARα targets was rather small and could only be made visible by performing whole genome expression profiling. Our results are in agreement with previous studies that have found a reduction in liver triglycerides following exercise training. As an explanation, these studies have hinted at reduced fatty acid uptake [17], increased mitochondrial oxidation and PPARα expression [18, 20] and reduced lipogenesis [18–20].

As an alternative explanation, we cannot exclude that every single exercise bout caused a very small decrease in the liver triglyceride pool, cumulatively causing the 30 % decrease in liver fat after 3 weeks of exercise training. Although such a minimal change in liver fat by a single exercise bout cannot be measured, it may be indirectly reflected in induction or suppression of certain metabolic pathways. We found that a single exercise bout significantly reduced expression of the lipogenic genes Srebf1 and Gpam. In line with this result, a single aerobic exercise bout performed after a carbohydrate-rich meal was found to significantly reduce hepatic triglyceride synthesis and *de novo* lipogenesis in human subjects [34].

Whereas liver triglycerides were reduced following 3 weeks of exercise training, liver glycogen content was significantly increased by exercise training. Liver and skeletal muscle are two major repositories that stores glucose in the form of glycogen. Liver glycogen levels are correlated with oxidative capacity of muscle, and are therefore an important determinant of exercise capacity in rodents [35]. While liver glycogen levels are comparable in human and mice, muscle glycogen levels are ~10 fold lower in mice compared to human [36]. Accordingly, the increase in liver glycogen may represent an adaptive response to regular exercise similar to the glycogen supercompensation observed in human muscle. The observed increase in liver glycogen levels may be explained by the reduction in AMPK phosphorylation, one of the key regulators of glycogen synthase 2. AMPK has been shown to be able to phosphorylate glycogen synthase 2 and thereby reduce its affinity towards its substrates UDP-Glucose and Glucose-6-P [37]. Induction of glycogen synthase 2 may be further mediated by increased gene expression. In contrast to liver glycogen, muscle glycogen content was reduced in the trained mice. The reason is unclear but may be due to an adaptive shift in fuel utilisation from glycogen to increased use of lipid fuels. Alternatively, it may be a carryover effect from the last exercise bout.

The transition from an exercise phase to a post-exercise phase is associated with a number of metabolic changes in the liver that may be influenced by AMPK. Activation of AMPK during exercise protects the cell against ATP depletion by stimulating processes such as fatty acid oxidation and suppressing ATP-utilizing lipogenic pathways. Accordingly, the observed decrease in hepatic AMPK activity in the trained mice may represent a transient physiological change to favour anabolic processes during the post-exercise phase. Along these lines, a time-dependent reduction in liver AMPK activity lasting up to 24 h has been reported during a similar physiological situation of fasting and refeeding [38].

Regular exercise is known to lower circulating triglyceride levels [39], likely due to improved triglyceride clearance mediated by elevated lipoprotein lipase activity in muscle [40]. The triglyceride-lowering effect is considered as an acute response to recent exercise rather than a long term training adaptation [41]. Previously, we found that a single 90 min treadmill exercise bout significantly reduced plasma triglyceride levels in mice, which was opposed by the lipoprotein lipase inhibitor ANGPTL4 [42]. The acute effect of exercise on plasma triglycerides rapidly reverses with detraining, which may explain the lack of difference in plasma triglycerides between trained and untrained mice. Alternatively, the lack of difference in triglycerides may be because the mice were not fasted at the time of sacrifice.

Several different animal models for hepatic steatosis are routinely used in NAFLD research. These models each have their specific advantages and disadvantages, and vary in their mechanistic and phenotypical resemblance to human NAFLD [15]. The animal model used in our study was specifically chosen to avoid the potential confounding of obesity and exercise-induced fat loss. By feeding a diet rich in sucrose but deficient in choline mice develop mild steatosis as shown by increased liver fat accumulation and liver enzymes. The likely mechanism is a combination of the lipogenic effect of dietary sucrose coupled to the influence of choline deficiency on lipoprotein metabolism and oxidative stress [43].

The use of non-obese mice could also be considered a limitation since NAFLD in humans is often, though not exclusively, accompanied by obesity. Previous studies in different mouse models of NAFLD including diet-induced obese mice confirm the suppressive effect of exercise training on liver triglycerides and also point to reduction of hepatic inflammation and fibrosis [16–23]. It should be noted, however, that the reduction in liver triglycerides in some studies may be at least partly caused by reduced adiposity following exercise training [16–21], which makes it difficult to make an appropriate comparison with our study. In our study, exercise training neither influenced bodyweight nor adipose tissue weight.

## Conclusions

The results of our study indicate that treadmill-based exercise training for three weeks reduced liver fat accumulation in mice without causing any changes in adipose tissue mass. Exploration of the potential underlying mechanism did not reveal a significant effect of exercise training on liver triglyceride secretion, *de novo* lipogenesis, or lipolysis/autophagy. Instead, our data point to a weak stimulatory effect of exercise training on PPARα-mediated regulation of oxidative metabolism. Taken together, our study suggests that the lowering of hepatic steatosis by repeated exercise is probably due to activation of fuel oxidation pathways in liver.

## Abbreviations

NAFLD: Non-alcoholic fatty liver disease
SECDD: Sucrose enriched choline deficient diet
DNL: De novo lipogenesis
AMPK: AMP-activated protein kinase
